# Supraspliceosomes at Defined Functional States Portray the Pre-Assembled Nature of the Pre-mRNA Processing Machine in the Cell Nucleus

**DOI:** 10.3390/ijms150711637

**Published:** 2014-06-30

**Authors:** Hani Kotzer-Nevo, Flavia de Lima Alves, Juri Rappsilber, Joseph Sperling, Ruth Sperling

**Affiliations:** 1 Department of Genetics, the Hebrew University of Jerusalem, Jerusalem 91904, Israel; E-Mail: hanikotzer@hotmail.com; 2 Wellcome Trust Centre for Cell Biology, University of Edinburgh, Edinburgh EH9 3JR, UK; E-Mails: flaves12@gmail.com (F.L.A.); juri.rappsilber@ed.ac.uk (J.R.); 3 Institute of Bioanalytics, Department of Biochemistry, Technische Universität Berlin, Berlin 13353, Germany; 4 Department of Organic Chemistry, the Weizmann Institute of Science, Rehovot 76100, Israel; E-Mail: j.sperling@weizmann.ac.il

**Keywords:** pre-mRNA splicing, specific supraspliceosomes, U snRNPs, PP7-tagged supraspliceosomes

## Abstract

When isolated from mammalian cell nuclei, all nuclear pre-mRNAs are packaged in multi-subunit large ribonucleoprotein complexes—supraspliceosomes—composed of four native spliceosomes interconnected by the pre-mRNA. Supraspliceosomes contain all five spliceosomal U snRNPs, together with other splicing factors, and are functional in splicing. Supraspliceosomes studied thus far represent the steady-state population of nuclear pre-mRNAs that were isolated at different stages of the splicing reaction. To analyze specific splicing complexes, here, we affinity purified *Pseudomonas aeruginosa* phage 7 (PP7)-tagged splicing complexes assembled *in vivo* on Adenovirus Major Late (AdML) transcripts at specific functional stages, and characterized them using molecular techniques including mass spectrometry. First, we show that these affinity purified splicing complexes assembled on PP7-tagged AdML mRNA or on PP7-tagged AdML pre-mRNA are assembled in supraspliceosomes. Second, similar to the general population of supraspliceosomes, these defined supraspliceosomes populations are assembled with all five U snRNPs at all splicing stages. This study shows that dynamic changes in base-pairing interactions of U snRNA:U snRNA and U snRNA:pre-mRNA that occur *in vivo* during the splicing reaction do not require changes in U snRNP composition of the supraspliceosome. Furthermore, there is no need to reassemble a native spliceosome for the splicing of each intron, and rearrangements of the interactions will suffice.

## 1. Introduction

The maturation of all pre-mRNA expressed from human protein-coding genes requires the processing of the pre-mRNA to an mRNA that can be transported from the nucleus to the cytoplasm to encode for proteins. The processing steps include capping of the 5' end, processing of the 3' end, editing and splicing. Splicing of the pre-mRNA molecule occurs within the spliceosome, a huge ribonucleoprotein (RNP) complex. The accuracy and efficiency of pre-mRNA splicing is attributed to a number of trans-acting factors, which include the spliceosomal uridine-rich small nuclear ribonucleoprotein complexes (U1, U2, U4, U5, and U6 snRNPs) and several non snRNP protein splicing factors, as well as to *cis*-acting sequence elements. The latter include 5' and 3' splice sites, a branch point, and a polypyrimidine tract. Splicing enhancers and silencers are additional control elements, which play an important role in both constitutive and alternative splicing by interacting with splicing factors, such as the Serine/Arginine (SR) rich protein family [1].

Studies *in vitro* have shown that the assembly of the spliceosome occurs in a stepwise manner [2,3,4]. The U snRNPs are central components of the spliceosome. They participate in splice-site recognition and have an essential function in splicing through cooperative RNA:RNA interactions between the snRNAs and the pre-mRNA [2,3,5]. The assembly of the spliceosome involves an intricate series of interactions between the five major U snRNPs, as well as with a number of non-snRNP splicing factors, which are dynamically recruited to the spliceosome when an exogenous pre-mRNA is added to a nuclear extract. A pre-catalytic complex involves the five-spliceosomal U snRNPs, yet the splicing active complex contains U2.U5/U6 snRNPs [1].

Regulated splicing, as well as constitutive splicing, operates through the combinatorial interplay of positive and negative regulatory signals present in the pre-mRNA, which are recognized by trans-acting factors. The most studied of the latter are members of the hnRNP and SR protein families. SR proteins, characterized by serine and arginine-rich protein motive, bind RNA through their RNA recognition motives (RRMs), while their SR domain appears to enable protein–protein and protein–RNA interactions during the splicing reaction. SR proteins show distinct RNA binding specificities for exonic splicing enhancers (ESEs), and multiple binding sites for several SR proteins are found in the same exon [6,7,8]. The hnRNP proteins are a diverse group of RNA binding proteins, with an RNA-binding domain (RBD). They are associated with RNA Pol II transcripts and play a key role in regulation of pre-mRNA splicing [9]. The high fidelity of exon recognition is thus achieved by the combination of multiple weak protein:protein, protein:RNA, and RNA:RNA interactions.

The composition of splicing complexes assembled *in vitro* was analyzed by mass spectrometry [1,10,11], mainly of affinity purified RNA-tagged spliceosomes. These studies showed over hundred proteins that are associated with the *in vitro* assembled spliceosome, and reported changes in protein composition that occurred during the splicing reaction. Mass spectrometry analysis of the general population of splicing complexes isolated from nuclei and co-immunoprecipitated by anti-Sm MAbs revealed 177 polypeptides [12].

When isolated from mammalian cell nuclei, Pol II transcripts are found assembled in huge (21 MDa) nuclear RNP particles termed supraspliceosomes. The entire repertoire of nuclear pre-mRNAs, independent of their length or the number of introns they contain, appear to be assembled in splicing-active supraspliceosomes [13]. Supraspliceosomes harbor all five spliceosomal U snRNPs and splicing factors [14,15]. In addition to the constitutive splicing factors, a number of splicing regulatory factors were found to be predominantly associated with supraspliceosomes. These include all phosphorylated SR proteins [16]; the splicing regulatory factor hnRNP G [17]; and the alternative splicing factors RBM4 and WT1, which co-interact to influence alternative splicing [18], and the regulatory splicing factor ZRANB2 [19]. Supraspliceosomes also harbor other components of pre-mRNA processing, such as the cap-binding proteins, components of the 3' end processing activity [20], and the editing enzymes ADAR1 and ADAR2 [21]. Taken together, these observations support the view that the supraspliceosome is the nuclear pre-mRNA processing machine.

Structural studies revealed that the supraspliceosome is composed of four apparently similar splicing active substructures—native spliceosomes—each resembling an *in vitro* assembled spliceosome, which are connected by the pre-mRNA [15,22,23,24,25,26]. The 3-D structure of the native spliceosome was determined by the cryo-electron microscopy (cryo-EM) single particle technique at a resolution of 20 Å, and revealed an elongated globular particle composed of a large and a small subunit [25]. Within the supraspliceosome, the native spliceosomes are arranged such that their small subunits reside in its center. This configuration allows communication between the native spliceosomes, which is a crucial element for regulated splicing and for quality control of the resulting mRNAs [15,26].

Previous studies of supraspliceosomes analyzed the steady state population of nuclear complexes assembled on the general population of Pol II transcripts, having different sequences and number of introns and likely assembled at different stages of the splicing reaction. Here we focused on analysis of splicing complexes assembled on a specific transcript at a defined functional state. For this aim we affinity purified splicing complexes assembled on transcripts harboring a *Pseudomonas aeruginosa* phage 7 (PP7)-tag, which we inserted in the 3'UTR or in the intron. We show that the affinity purified splicing complexes, which were isolated via their PP7-tag, are assembled in supraspliceosomes. We further show that supraspliceosomes assembled on a specific mRNA or pre-mRNA contain all five spliceosomal U snRNPs. We can, thus, conclude that supraspliceosomes isolated from mammalian cell nuclei have all five spliceosomal U snRNAs associated with them at all splicing stages. However, we do observe some differences in the protein composition of supraspliceosomes from the different splicing stages.

## 2. Results and Discussion

The structural and functional analysis of supraspliceosomes performed so far was of the steady state population of complexes assembled on the general population of nuclear pre-mRNAs, each harboring a different number of introns and is likely to represent a different step of the splicing process [13,14,15,22,24,27]. Importantly, solving the structure of the supraspliceosome, at 20 Å resolution [25], and the fact that the scanning transmission EM (STEM) mass measurements revealed a relatively uniform mass for the supraspliceosome (21.1 ± 1.6 MDa; *n* = 400) [24], likely reflects the presence of a general basic structure—the supraspliceosome—that at this resolution is similar for the different transcripts. During the steps of the splicing reaction, changes in RNA–RNA, protein–RNA and protein–protein interaction occur [5,28]. In order to study how the changes in these internal interactions are reflected in the supraspliceosome, we affinity purified and analyzed supraspliceosomes assembled on specific transcripts, isolated at defined functional states (pre-mRNA or mRNA). The isolation was based on preparation of stable cell lines transcribing specific RNA-tagged transcripts, and the isolation of splicing complexes assembled on transcripts at defined functional states was based on mutations of genes encoding specific pre-mRNAs.

### 2.1. Generation of Stable Cell Lines Expressing Pseudomonas aeruginosa Phage 7 (PP7)-Tagged Adenovirus Major Late (AdML) Transcripts

For this approach we used the “RNA Affinity in Tandem” system [29]. This RNA tag-based method uses the *Pseudomonas aeruginosa* phage 7 (PP7) RNA binding site to tag the RNA, and was developed for affinity purification of endogenously assembled RNP complexes. Affinity purification is mediated by a recombinant PP7 coat protein (PP7CP), as an *N*-terminal fusion to two Protein A domains (ZZ), with an intervening linker for cleavage by Tobacco Etch Virus (TEV) protease (ZZTEVPP7CP). The PP7CP binds to the RNA tag with high affinity and high selectivity under various conditions including physiological conditions [30,31]. Recovery of the coat protein and associated RNPs was achieved using IgG agarose beads and elution from washed resin by addition of TEV protease. As the yield of this method was found to be higher than the MS2/R17 system [29], it seemed advantageous for our study.

**Figure 1:**
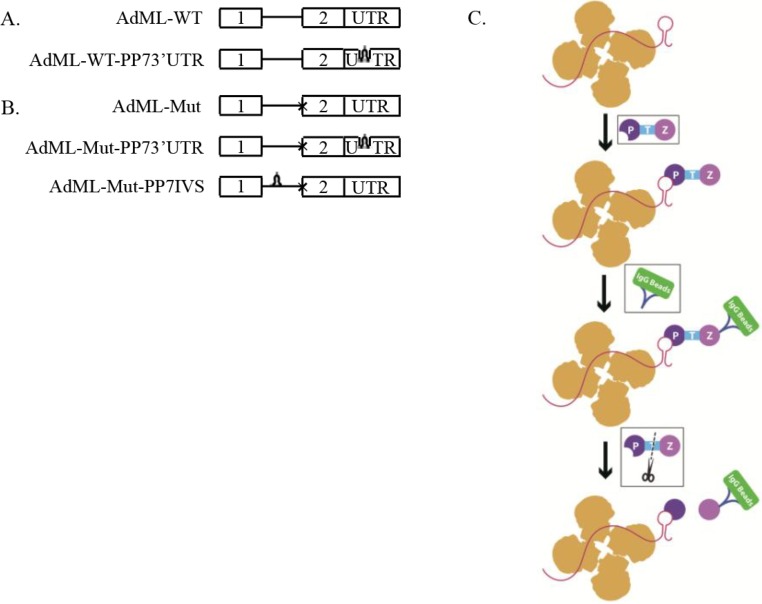
Scheme of the affinity purification of specific supraspliceosomes. (**A**,**B**) Schemes of AdML constructs used for preparation of stable cell lines, each expressing one of the constructs used for the affinity purification of specific supraspliceosomes at defined splicing stages. (**A**) Constructs expressing AdML-WT; (**B**) constructs expressing AdML-Mut, having a mutated 3' splice site, designated “*x*”. The upper schemes represent the control construct without PP7 tag. The middle scheme of (**B**) and the lower scheme of (**A**) represent the constructs harboring the PP7 tag at the 3'UTR. The lower scheme of (**B**) represents the construct harboring the PP7 tag at the intron. Open boxes represent exons, lines represent introns, and stem-loops represent the PP7 tag; (**C**) Scheme of the affinity purification process of specific splicing complexes assembled on a specific transcript. First, nuclear supernatants enriched in splicing complexes (scheme in yellow) are extracted from HeLa cell-lines expressing the respective AdML construct. A fusion protein ZZTEVPP7CP (Purple; Z: ZZ; T: TEV; P: PP7CP) is then added, and the PP7CP part bind the PP7 tag. Next, IgG agarose beads (Blue, Green, respectively) were added for binding of the ZZ part of the fusion protein to the antibody, followed by sedimentation and washing of the beads. The elution of splicing complexes (Yellow) assembled on the respective specific transcript harboring the PP7 tag, and bound to the PP7CP, is performed by digestion with TEV protease (Black scissors).

We, thus, prepared a number of constructs of Adenovirus Major Late (AdML) harboring a PP7 tag, and cloned them into pcDNA3 vector (Figure 1). Two wild type (WT) AdML constructs were prepared (Figure 1A): one with no tag (AdML-WT), and a second, with the tag at the 3'UTR (AdML-WT-PP73'UTR). We also prepared three Mutant (Mut) clones that are inhibited after the first step of splicing (Figure 1B). One, mutant construct without a tag (AdML-Mut); Second, a Mut AdML construct with a PP7 tag at the 3'UTR (AdML-Mut-PP73'UTR); and a third construct of Mut AdML with the PP7 tag at the intron (AdML-Mut-PP7IVS). All constructs were confirmed by sequencing. We next prepared five HeLa stable cell lines, each stably expressing one of the above constructs, respectively.

### 2.2. Affinity Purification of Complexes Assembled on AdML-WT Transcripts

Affinity purification of splicing complexes assembled on PP7-tagged AdML-WT transcripts was performed as described in the Experimental Section (see schematics in Figure 1C). Briefly, a HeLa cell line stably expressing AdML-WT with a PP7 tag at the 3'UTR (AdML-WT-PP73'UTR) was grown in tissue culture, and nuclear supernatants were prepared as described [15]. Next, the nuclear supernatants were incubated with ZZTEVPP7CP, and the reaction mixture was further incubated with Rabbit IgG beads, followed by washing of the beads. For elution of the splicing complexes the reaction was incubated overnight, with TEV protease. After pelleting the beads, the supernatant was collected and analyzed. As control, we performed, in parallel, the whole procedure with nuclear supernatants prepared from a HeLa cell line stably expressing AdML-WT without a tag.

**Figure 2:**
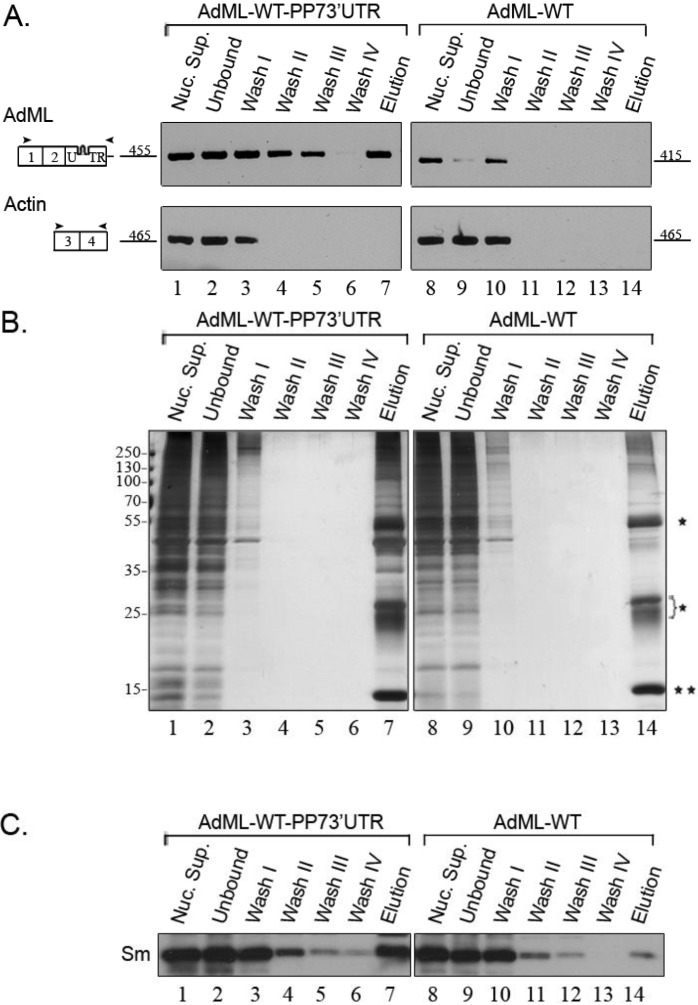
Analysis of affinity purified splicing complexes assembled on AdML-WT-PP73'UTR. Nuclear supernatants prepared from cell lines stably expressing the AdML-WT-PP73'UTR transcript, harboring the PP7 tag at the 3'UTR (**left**), or AdML-WT transcript without the tag (**right**), were affinity purified. Aliquots from the different steps of the affinity purification procedure were analyzed by RT-PCR (**A**); silver staining of SDS-PAGE (**B**); and WB (**C**). Lanes 1 and 8, Nuclear Supernatant (Nuc. Sup., 10%); Lanes 2 and 9, material not bound to the beads (Unbound); Lanes 3–6 and 10–13, washes; Lanes 7 and 14, elution. (**A**) RT-PCR products were electrophoresed on 1% agarose gel. Their identity is given on the left, open boxes represent exons, arrowheads represent the PCR primers. The expected size of each PCR product (*nt*) is given adjacent to its location on the left; (**B**) Proteins from the different steps of the affinity purification procedure were electrophoresed on 10% polyacrylamide gel and the gel was silver stained. Protein size marker is given on the left. *, heavy and light chains of the IgG antibody used for the affinity purification procedure; **, PP7CP protein; (**C**) Proteins from the different steps of the affinity purification procedure were electrophoresed on 12% polyacrylamide gel, transferred to a Nitrocellulose membrane and analyzed by WB using an anti-Sm antibody.

The specificity of the purification was first analyzed by RT-PCR (Figure 2A). As can be seen, mature AdML-WT RNA is eluted when AdML-WT-PP73'UTR is affinity purified, while no AdML RNA is eluted when nuclear supernatants prepared from a stable cell line expressing AdML-WT that lacks the PP7-tag are going through the same steps. As expected, actin transcripts that do not harbor the PP7 tag are not eluted. We also examined the protein profile of the eluted material by SDS-PAGE and silver staining (Figure 2B). While a large number of proteins are found in the affinity purified complexes assembled on AdML-WT-PP73'UTR, in the elution of AdML-WT we see mainly the light and heavy chains of IgG, originating from the beads, as well as the PP7CP. We next checked the affinity purification by Western Blot (WB), using antibodies against the Sm proteins, which are integral components of splicing complexes [4,20]. Figure 2C shows that Sm proteins are associated with the eluted AdML-WT-PP73'UTR splicing complexes, while only traces of Sm proteins are found in the control. We can, thus, conclude that we have affinity purified RNP complexes of the AdML-WT-PP73'UTR transcript.

### 2.3. Affinity Purified PP7-Tagged AdML-WT Transcripts Are Assembled in Supraspliceosomes

**Figure 3:**
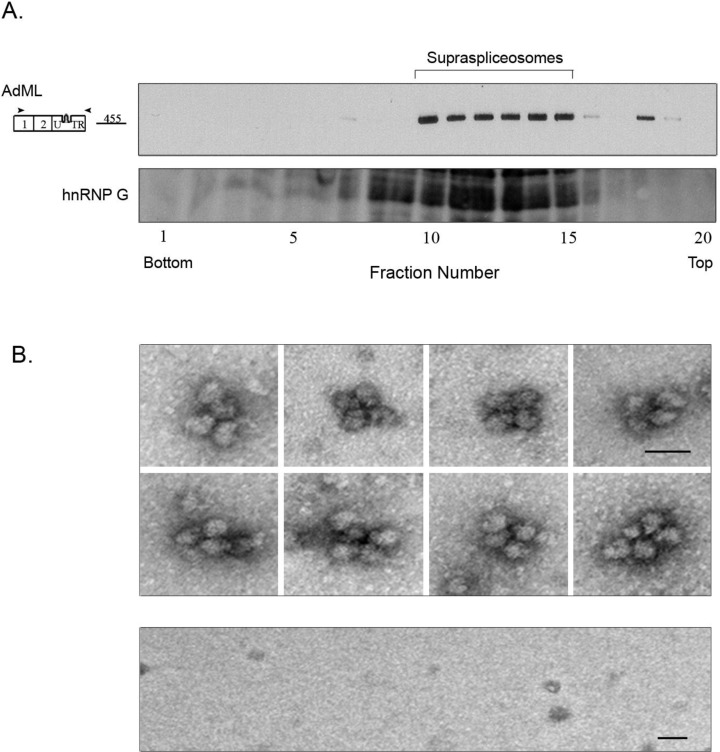
The affinity purified splicing complexes assembled *in vivo* on AdML-WT-PP73'UTR transcripts are supraspliceosomes. (**A**) **Upper** panel: Affinity purified splicing complexes assembled on the AdML-WT-PP73'UTR transcript, were fractionated in 10%–45% glycerol gradients, under the conditions used for fractionation of supraspliceosomes [15]. RNA was extracted from each fraction, analyzed by RT-PCR using the indicated AdML primer pair (arrowheads), and the amplified products were electrophoresed on 1% agarose gel. The size of the PCR product (*nt*) is given adjacent to its location, open boxes represent exons; **Lower** panel, Western Blot analysis of nuclear supernatants enriched for supraspliceosomes prepared from HeLa cells and fractionated in 10%–45% glycerol gradients. Aliquots from gradient fractions were analyzed by WB using anti-hnRNP G antibody. hnRNP G was previously shown to predominantly sediment with supraspliceosomes and to be associated with them [17]; (**B**) **Upper** panel, Gallery of electron micrographs of affinity purified supraspliceosomes assembled on AdML-WT-PP73'UTR transcript, visualized by EM, after negative staining with 1% uranyl acetate; **Lower** panel, Control, electron micrograph of eluted material from the control sample (AdML-WT transcript without the PP7 tag). The bar represents 50 nm.

In previous studies we isolated supraspliceosomes by centrifugation in glycerol/sucrose gradients, and showed that Pol II transcripts are individually assembled in supraspliceosomes that sediment at 200S in such gradients (reviewed in [13]). Since here we used a different protocol for isolation of splicing complexes assembled on PP7-tagged transcripts, namely by affinity purification of splicing complexes assembled on PP7-tagged transcripts, using recombinant PP7CP, we used glycerol gradient fractionation to determine the sedimentation velocity of the complexes in which the affinity purified AdML-WT-PP73'UTR transcripts are assembled. We, thus, loaded the affinity purified AdML-WT-PP73'UTR complexes on glycerol gradients and analyzed the distribution of AdML RNA across the gradient by RT-PCR. As can be seen in Figure 3A, AdML-WT-PP73'UTR transcripts were assembled in complexes that sediment at a sedimentation velocity that supraspliceosomes sediment, having a similar sedimentation to that of hnRNP G. It should be pointed out that we have previously shown that hnRNP G predominantly sediments with supraspliceosomes and is associated with them [17].

Further confirmation that affinity purified PP7-tagged AdML-WT-PP73'UTR transcripts are assembled in supraspliceosomes comes from EM visualization (Figure 3B). When aliquots from the eluted AdML-WT-PP73'UTR complexes were negatively stained and visualized by EM, the affinity purified splicing complexes of AdML-WT-PP73'UTR were observed assembled in supraspliceosomes, (Figure 3B, upper panel), similar in shape and dimensions to the 50 nm tetrameric quadrangular general population of supraspliceosomes [22,24]. In addition, the dimensions of the native spliceosomes within the supraspliceosome are similar to those found previously for the general population of native spliceosomes [24,25]. No supraspliceosomes were observed eluted from the control (Figure 3B, lower panel). We can thus conclude that AdML-WT-PP73'UTR transcripts are assembled in supraspliceosomes, and that the specific splicing complexes assembled on PP7-tagged AdML that were affinity purified by the PP7/PP7CP system are supraspliceosomes. Furthermore, the specific supraspliceosomes assembled on AdML-WT-PP73'UTR are supraspliceosomes assembled on mature polyadnylated AdML (unspliced RNA could not be detected) as can be seen in Figure 2A.

### 2.4. Affinity Purification of Supraspliceosomes Assembled on AdML-Mut Transcripts

**Figure 4:**
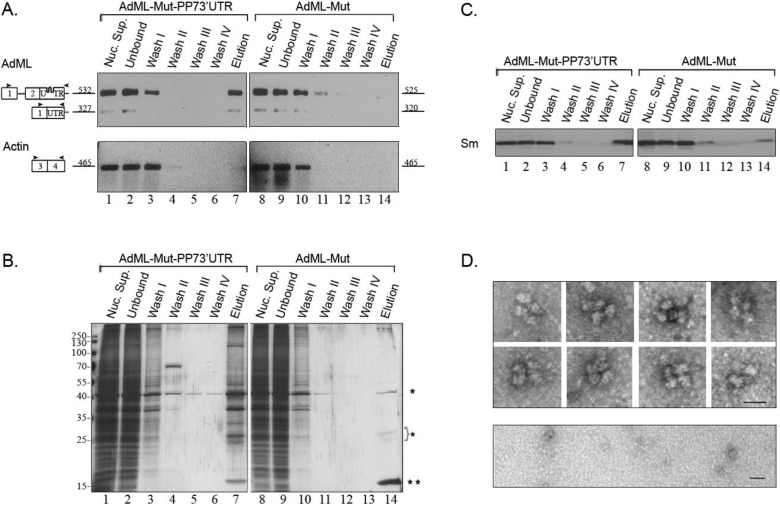
Analysis of affinity purified suprasliceosomes assembled on AdML-Mut-PP73'UTR. Nuclear supernatants prepared from cell lines stably expressing the AdML-Mut-PP73'UTR transcript, harboring the PP7 tag at the 3'UTR (**left**) or AdML-Mut transcript without the tag (**right**) were affinity purified. Aliquots from the different steps of the affinity purification procedure were analyzed by RT-PCR (**A**); silver staining of SDS-PAGE (**B**) and WB (**C**). Lanes 1 and 8, Nuclear Supernatant (Nuc. Sup., 10%); Lanes 2 and 9, material not bound to the beads (Unbound); Lanes 3–6 and 10–13, washes; Lanes 7 and 14, elution. (**A**) RT-PCR products were electrophoresed on 1% agarose gel. Their identity is given on the left, open boxes represent exons, line intron, arrowheads represent PCR primers. The expected size of each PCR product (*nt*) is given adjacent to its location on the left; (**B**) Proteins from the different steps of the affinity purification procedure were electrophoresed on 10% polyacrylamide gel and the gel was silver stained. Protein size marker is given on the left. *, heavy and light chains of the IgG antibody used for the affinity purification procedure. **, PP7CP protein; (**C**) Proteins from the different steps of the affinity purification procedure were electrophoresed on 12% polyacrylamide gel, transferred to a Nitrocellulose membrane and analyzed by WB using an anti-Sm antibody; (**D**) EM visualization. **Upper** panel, Gallery of electron micrographs of affinity purified supraspliceosomes assembled on AdML-Mut-PP73'UTR transcript, visualized by EM, after negative staining with 1% uranyl acetate; **Lower** panel, Control, electron micrograph of eluted material from the control sample (AdML-Mut transcript without the PP7 tag). The bar represents 50 nm.

A similar affinity purification procedure was used to isolate supraspliceosomes assembled on AdML-Mut-PP73'UTR transcripts. These transcripts harbor a mutated 3' splice site and cannot undergo the second step of splicing. RT-PCR analysis of the affinity purified complexes assembled on AdML-Mut-PP73'UTR revealed the specificity of isolation, as no AdML-Mut transcripts were observed in the control harboring no tag, and also no actin transcripts were eluted (Figure 4A). The specificity is also confirmed by silver staining of SDS-PAGE and by Western Blot with anti-Sm antibodies (Figure 4B,C, respectively). The eluted transcripts are mainly AdML-Mut pre-mRNA (Figure 4A). EM visualization of eluted complexes assembled on AdML-Mut-PP73'UTR transcripts revealed supraspliceosomes (Figure 4D, upper panel), similar to those observed for the WT transcripts and similar in shape and dimensions to the 50 nm tetrameric quadrangular general population of supraspliceosomes [22,24], while no distinct complexes were observed in the control (Figure 4D, lower panel). Thus, affinity purification of nuclear complexes assembled on AdML-Mut-PP73'UTR transcripts yielded supraspliceosomes assembled on AdML-Mut pre-mRNA, having a PP7 tag at the 3'UTR.

**Figure 5:**
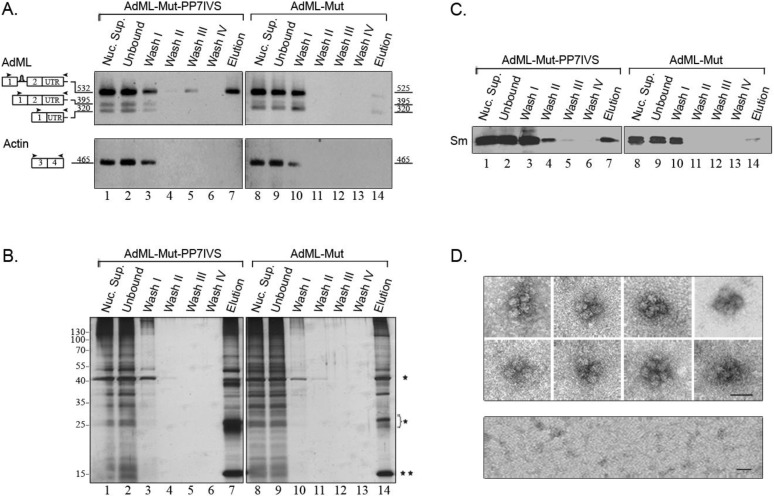
Analysis of affinity purified suprasliceosomes assembled on AdML-Mut-PP7IVS. Nuclear supernatants prepared from cell lines stably expressing the AdML-Mut-PP7IVS transcript, harboring the PP7 tag at the intron (**left**) or AdML-Mut transcript without the tag (**right**) were affinity purified. Aliquots from the different steps of the affinity purification procedure were analyzed by RT-PCR (**A**); silver staining of SDS-PAGE (**B**) and WB (**C**). Lanes 1 and 8, Nuclear Supernatant (Nuc. Sup., 10%); Lanes 2 and 9, material not bound to the beads (Unbound); Lanes 3–6 and 10–13, washes; Lanes 7 and 14, elution. (**A**) RT-PCR products were electrophoresed on 1% agarose gel. Their identity is given on the left, open boxes represent exons, line intron, arrowheads represent PCR primers. The expected size of each PCR product (*nt*) is given adjacent to its location on the left; (**B**) Proteins from the different steps of the affinity purification procedure were electrophoresed on 10% polyacrylamide gel and the gel was silver stained. Protein size marker is given on the left. *, heavy and light chains of the IgG antibody used for the affinity purification procedure. **, PP7CP protein; (**C**) Proteins from the different steps of the affinity purification procedure were electrophoresed on 12% polyacrylamide gel, transferred to a Nitrocellulose membrane and analyzed by WB using an anti-Sm antibody; (**D**) EM visualization. **Upper** panel, Gallery of electron micrographs of affinity purified supraspliceosomes assembled on AdML-Mut-PP7IVS transcript, visualized by EM, after negative staining with 1% uranyl acetate. **Lower** panel, Control, electron micrograph of eluted material from the control sample (AdML-Mut transcript without the PP7 tag). The bar represents 50 nm.

We next affinity purified splicing complexes assembled on AdML-Mut-PP7IVS. Affinity purification of splicing complexes assembled on AdML-Mut-PP7IVS is advantageous because it should enable isolation of complexes that do not have contamination of mature transcripts in them. This is because the presence of the PP7 tag in the intron should enable affinity purification only of complexes assembled on intron containing transcripts. As can be seen in Figure 5A, despite the mutation at the 3' splice site, low levels of mature RNA, one with skipping of exon 2 and another one with use of a cryptic 3' splice site were observed in the nuclear supernatants prepared from this cell line. However, the eluted material is devoid of mature RNA and only pre-mRNA is observed. The specificity of the affinity purification is demonstrated by the RT-PCR analysis (Figure 5A), by silver staining of SDS-PAGE (Figure 5B), and by the WB with anti-Sm antibodies (Figure 5C). EM visualization revealed supraspliceosomes similar to those observed for the WT transcripts (Figure 5D, upper panel), and similar in shape and dimensions to the 50 nm tetrameric quadrangular general population of supraspliceosomes [22,24]. Additionally, the dimensions of the native spliceosomes within the supraspliceosomes are similar to those found for the WT native spliceosomes. Also, the dimensions of the native spliceosomes within the supraspliceosomes are similar to those found previously for the general population of native spliceosomes [24,25], while no distinct complexes were observed in the control (Figure 5D, lower panel).

We can conclude that affinity purified splicing complexes assembled on either AdML-WT or AdML-Mut transcripts are assembled in supraspliceosomes. AdML-WT supraspliceosomes are assembled on mature RNA, and ADML-Mut supraspliceosomes are assembled on pre-mRNA.

Each supraspliceosome is assembled on one pre-mRNA, as revealed by EM visualization of supraspliceosomes reconstituted from native spliceosomes and pre-mRNA tagged at its 3' end with one Nanogold particle (1.4 nm) [32]. The four native spliceosomes of the supraspliceosome are connected by the pre-mRNA. In this configuration, the supraspliceosome acts as a multiprocessor that can simultaneously splice four introns, not necessarily in a consecutive manner [13]. As all RNA Pol II transcripts are assembled in supraspliceosomes, we proposed that splicing of multi-intronic transcripts is facilitated by translocation of the pre-mRNA through the complex [15,22]. In this “rolling model” after processing of four introns, the pre-mRNA rolls in to place a new subset of introns in the right position that allows their processing. The present study demonstrates that in the cell nucleus, transcripts having one intron are also assembled and processed in supraspliceosomes. This is in accordance with previous experiments with reconstituted supraspliceosomes, which showed that pre-mRNAs with less than four introns are also assembled in supraspliceosomes. Likely, the interactions of such RNA with the native spliceosomes are sufficient to hold the supraspliceosome structure together [15].

### 2.5. Supraspliceosomes Isolated from Cell Nuclei Are Assembled with All Five Spliceosomal U snRNPs during the Steps of the Splicing Reaction

**Figure 6:**
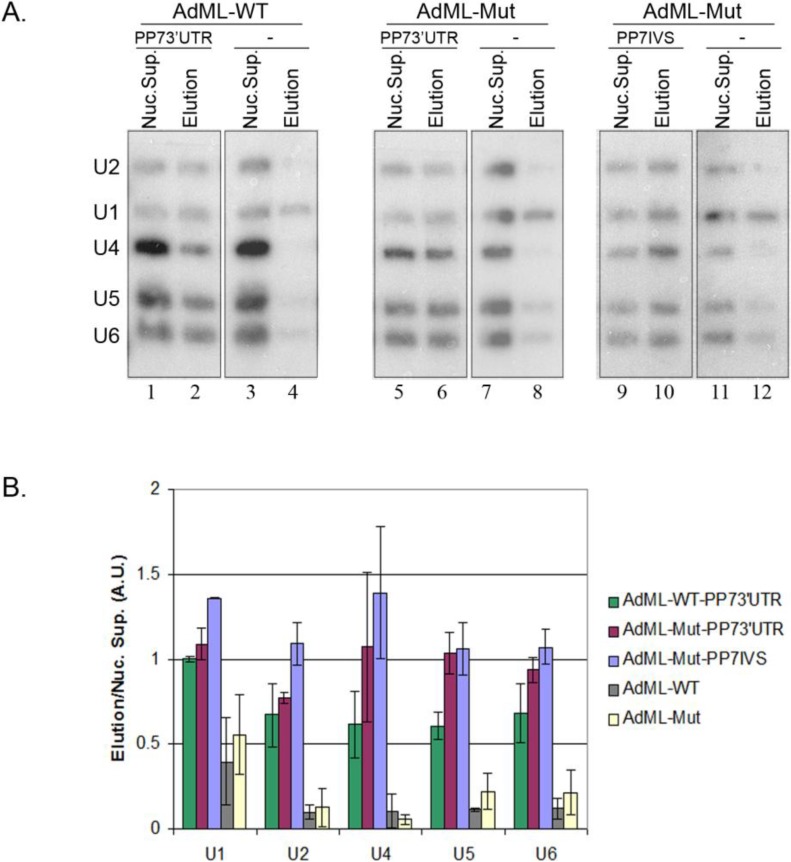
The five spliceosomal U snRNPs are associated with supraspliceosomes assembled *in vivo* at all splicing stages. (**A**) Northern blot analysis with probes directed against the five spliceosomal U snRNAs was performed on RNA extracted from nuclear supernatants and from affinity purified splicing complexes prepared from HeLa cell-lines expressing the AdML constructs either with the PP7 tag or without it (−), as indicated. **Left**, the AdML-WT-PP73'UTR transcript; **middle**, the AdML-Mut-PP73'UTR transcript; **Right**, the AdML-Mut-PP7IVS transcript. RNA was extracted either from the nuclear supernatant (Nuc. Sup.), or from the affinity purified samples (Elution). Aliquots were electrophoresed on 7 M urea/10% polyacrylamide gel, transferred to a nylon membrane, and hybridized with [α-^32^P] UTP-labeled RNA probes against the U1, U2, U4, U5, and U6 snRNAs, as described [15,16]. The identity of the U snRNA probes is given on the left; (**B**) Quantification of the Northern analyses. For each U snRNA molecule, the ratio between the signal of the eluted material and the relevant nuclear supernatant is shown. Each bar represents an average of two experiments; standard deviation is shown on the graph.

We have previously shown that supraspliceosomes isolated from mammalian cell nuclei have all five spliceosomal U snRNAs associated with them [14,15,16]. Furthermore, native spliceosomes, the subunits from which the supraspliceosome is composed, also harbor all five spliceosomal U snRNAs [15]. Our previous studies dealt with the steady state population of Pol II transcripts that differ in the number of their introns and exons and were at different stages of the splicing reaction. However, since in this study we affinity purified specific supraspliceosomes at defined functional states, we can analyze now the U snRNA composition of these specific supraspliceosomes. To this end, nuclear supernatants were prepared from each of the stable cell lines expressing: AdML-WT-PP73'UTR, AdML-Mut-PP73'UTR, AdML-Mut-PP7IVS, and from the control cell lines expressing AdML-WT, and AdML-Mut. Next, each of the nuclear supernatants was incubated with PP7CP and underwent through the affinity purification steps described above. After the affinity purification step, RNA was extracted from each of the nuclear supernatants and from each of the affinity purified samples prepared from it, and the RNA was electrophoresed on denaturing polyacrylamide gels. The gels were transferred to charged nylon membrane and hybridized simultaneously with [^32^P]RNA probes complementary to each of the five-spliceosomal U snRNAs. As can be seen in Figure 6A, all five spliceosomal U snRNAs were found associated with AdML-WT-PP73'UTR supraspliceosomes that are assembled on mature RNA (Lane 2), and on AdML-Mut-PP73'UTR and AdML-Mut-PP7IVS supraspliceosomes that are assembled on AdML pre-mRNA (Lanes 6 and 10, respectively). In the respective control samples that lack the PP7 tag (Lanes 4, 8, and 12), very low levels of U snRNAs are observed with some non specific binding of U1 snRNA, which is known to be in access in the cell nucleus [33]. Figure 6B presents averaging of the results of two experiments, where the *y*-axis depicts the ratio of the eluted material to the relevant nuclear supernatant. We can, therefore, conclude that supraspliceosomes isolated from mammalian cell nuclei have all five spliceosomal U snRNAs associated with them at all splicing stages.

Studies of spliceosome assembly *in vitro* revealed a stepwise assembly of spliceosomal U snRNPs during the splicing reaction [3,5], whereas the assembly of newly transcribed pre-mRNAs into supraspliceosomes *in vivo* appeared to involve pre-formed complexes [15,34,35]. Furthermore, the assembly pathway *in vitro* is characterized by major changes in composition (e.g., only three of the five spliceosomal snRNPs are part of the active C complex [36]). On the other hand, we have previously shown that both the general population of supraspliceosomes and native spliceosomes contained all five spliceosomal snRNPs [15]. These findings were strengthened by the isolation of a functional “penta-snRNP” complex from yeast [34], and highlight the important role of large, pre-formed, complexes in pre-mRNA splicing *in vivo*. The present study that analyses the U snRNP composition of specific supraspliceosomes, of defined sequence and of defined functional state, revealed that the supraspliceosome is assembled with all five spliceosomal U snRNPs (Figure 6). The apparent discrepancy between the notion of a stepwise assembly pathway of the spliceosome *in vitro* and the occurrence of a pre-formed splicing complex *in vivo*, has been explained by a “holospliceosome” model, in which the sequential complexes represent ordered modulations within the *in vivo* assembled spliceosome without the loss of components [3]. It has also been pointed out that such distinct complexes, which represent intermediate states in spliceosome assembly *in vitro*, may not occur *in vivo* [34,37].

The assembly pathways of supraspliceosomes *in vivo* and that of spliceosomes assembled *in vitro* differ in the sense that the former occurs co-transcriptionally in the nucleus, whereas the assembly process *in vitro* occurs when a full-length pre-mRNA is interacting with the spliceosomal components present in a nuclear extract. We suggest that these two likely distinct pathways may lead to different local minima in the respective free energy profiles, which may result in the assembly of slightly different complexes. Alternatively, the observed changes in composition between intermediate complexes assembled *in vitro*, which are not found in supraspliceosomes assembled *in vivo*, may be due to the lack of specific components in the *in vitro* assembled complexes. Such missing components might help keep the *in vivo* assembled complexes intact. (e.g., *in vitro* assembled splicing complexes were usually isolated in the presence of heparin, which could have caused partial dissociation of components [38,39]). The assembly pathway of spliceosomes *in vivo* and *in vitro* is an important issue in understanding splicing and its regulation [40]. Our results contribute to a better understanding of the spliceosome assembly pathway *in vivo.*

### 2.6. Protein Analysis of Affinity Purified Supraspliceosomes

Analyses of the protein composition of spliceosomes were conducted thus far mainly of different spliceosome complexes assembled *in vitro* during the assembly pathway of the spliceosome and the splicing reaction [1,10,36,38,41,42], and of the general population of supraspliceosomes co-immunoprecipitated using anti-Sm MAbs [12]. Here, we were interested in analyzing the protein composition of specific supraspliceosomes at defined splicing stages. We, therefore, analyzed the affinity purified supraspliceosomes assembled on AdML-WT-PP73'UTR, AdML-Mut-PP73'UTR and AdML-Mut-PP7IVS transcripts by liquid chromatography-coupled mass spectrometry (Table 1, Table 2 and Table S1). Three analyses of the affinity purified supraspliceosomes assembled on mature mRNA (AdML-WT-PP73'UTR), and two analyses of the supraspliceosomes assembled on pre-mRNA (on the AdML-Mut transcripts) were performed. The analyses revealed an average of 275 protein groups, higher amount than the 177 protein groups detected previously for the analysis of a general population of supraspliceosomes purified from HeLa cells [12]. The difference in number of proteins is likely a result of the different purification methods and instrumentation used for analysis. We found that the location of the PP7 tag within the transcript, either in the 3'UTR or the intron, had no effect, since no apparent differences were observed between the two AdML-Mut supraspliceosome populations. Similarly, the three AdML-WT assembled supraspliceosome populations gave a similar protein inventory; therefore, the results of the AdML-Mut supraspliceosomes analyses were combined for presentation, and so were the results for the AdML-WT supraspliceosomes. The main protein groups are shown in Table 1.

**Table 1:** Mass Spectrometry (MS) analyses.

Symbol	Protein	kDa	AdML-WT	AdML-Mut
U1 snRNP				
SNRNP70	U1-70 k	51.5	+	
U2 snRNP				
SNRP A1	U2A'	28.4	+	
SNRP B2	U2B''	25.5	+	
SF3A1	SF3a120	88.9	+	
SF3A2	SF3a66	49.3	+	
SF3A3	SF3a60	58.8	+	+
SF3B1	SF3b155	145.8	+	+
SF3B2	SF3b145	100.2	+	+
SF3B3	SF3b130	135.6	+	+
DDX46	PRP5	117.4	+	+
PHF5A	SF3b14b	12.4	+	+
U2 snRNP related				
U2AF2	U2AF65	53.5	+	+
U4/U6.U5 snRNP				
PRPF8	U5-220k	273.6	+	+
SNRNP200	U5-200k	244.5	+	+
EFTUD2	U5-116k	109.5	+	+
PRPF6	U5-102k	106.9		+
DDX23	U5-100k	93.2	+	+
PRPF3	U4/U6-90k	77.5	+	
PRPF4	U4/U6-60k	58.4	+	+
PRPF31	U4/U6.U5-61k	55.5	+	
Sm proteins				
SNRP B	Sm B/B'	24.6	+	+
SNRP D1	Sm D1	13.3	+	+
SNRP D2	Sm D2	13.5	+	
SNRP D3	Sm D3	13.9	+	+
SNRP E	Sm E	10.8	+	+
CK005235	Sm G Like	85.4	+	+
hPRP19/CDC5L complex				
PRPF19	PRPF19	55.2	+	+
CDC5L	CDC5	92.3	+	
HSPA8	HSP71	70.9	+	+
PLRG1	PRP45	57.2	+	
XAB2	SYF1	99.7	+	
AQR	Aquarius	156.8	+	
Splicing factors				
DHX15	DHX15	89.5	+	+
DDX39	DDX39	49.1	+	+
DDX5	DDX5 p68	69.1	+	+
FUS	FUS TLS	53.4	+	+
SFPQ	hPOMp100	68.7	+	+
TIA1	p40-TIA-1	31.6	+	
Splicing factors				
NONO	p54(nrb)	54.3	+	+
KHSRP	p75	73.1	+	
PABPC1	PABP1	70.7	+	+
PUF60	PUF60	59.9	+	
RBM4	RBM4	40.2	+	
TARDBP	TDP43	31.8	+	
DDX39B	UAP56	50.7	+	+
YBX1	YBX1	35.9	+	+
hnRNP proteins				
HNRNPA0	hnRNP A0	30.8	+	+
HNRNPA1	hnRNP A1	38.8	+	+
HNRNPA3	hnRNP A3	39.6	+	+
HNRNPA2B1	hnRNP A2/B1	37.4	+	+
HNRNPC	hnRNP C1/C2	33.7	+	+
RALY	hnRNP CL2	24.7	+	+
HNRNPD	hnRNP D	38.4	+	
HNRNPDL	hnRNP DL	46.4	+	+
PCBP1	hnRNP E1	37.5	+	+
PCBP2	hnRNP E2	33.9	+	+
HNRNPF	hnRNP F	45.7	+	+
RBMX	hnRNP G	42.3	+	+
HNRNPH1	hnRNP H1	49.2	+	+
HNRNPH2	hnRNP H2	49.3	+	+
HNRNPH3	hnRNP H3	36.9	+	+
PTBP1	hnRNP I	57.2	+	+
HNRNPK	hnRNP K	51	+	+
HNRNPL	hnRNP L	64.1	+	+
HNRNPM	hnRNP M	77.5	+	+
SYNCRIP	hnRNP Q	69.6	+	+
HNRNPR	hnRNP R	70.9	+	+
HNRNPU	hnRNP U	90.6	+	+
HNRNPUL1	hnRNP UL1	95.7	+	+
HNRNPUL2	hnRNP UL2	85.1	+	+
SR proteins				
SRSF1	SF2/ASF	28.3	+	+
SFR3	SRp20	19.3	+	+
SFR10	SFRS10	33.7	+	+
SRRM2	SRm300	299.6		+
SFRS11	SRp54	24.8		+
SRSF2	SRSF2	21.4		+
SRSF6	SRp55	39.4	+	+
SRSF7	SRSF7	18.8	+	+
3' polyA/processing factors				
NUD21	CPSF 25	26.2	+	+
3' polyA/processing factors				
CPSF7	CPSF 59	51.1	+	+
CPSF6	CPSF 68	52.3	+	+
CPSF3	CPSF 73	73.5	+	
CPSF2	CPSF 100	88.5	+	
CPSF1	CPSF 160	152	+	
CSTF2	CSTF64	46.7	+	
PABPN1	PABII	11	+	+
SYMPK	Symplekin	141.1	+	
Cap binding				
NCBP1	CBP80	91.8	+	
mRNA export and surveillance				
UPF1	UPF1	124.3	+	+
ALYREF	ALYREF	27.6	+	
MAGOHB	MAGOHB	17.3	+	
EIF4A3	eIF-4A-III	46.9	+	+
IGF2BP1	IMP-1	63.5	+	
RAN	RAN	24.4	+	+
XPO1	Exportin	123.4	+	
RANBP2	RANBP2	358.2	+	+
RANGAP1	RANGAP1	63.5	+	
DDX3X	DDX3X	73.2	+	+
RNA metabolism				
CSL4	CSL4	18.8	+	
DDX1	DBP-RB	82.4	+	+
DDX21	DDX21	87.3	+	+
DIS3	DIS3	109.1	+	
ELAVL1	HuR	39	+	+
LRPPRC	LRP 130	157.9	+	
ILF2	NF45	43.1	+	+
NF90	NF90	76	+	+
ILF3	NF110	95.3	+	
NCL	Nucleolin	76.6	+	+
RNA metabolism				
NPM1	Nucleophosmin	32.6	+	+
PABPC4	PABP-4	70.8	+	+
EXOSC2	RRP4	32.8	+	
SKIV2L2	SKIV2L2	117.8	+	
SRRT	SRRT	100.7	+	+
TIAL1	TIAL1	41.6	+	

**Table 2:** MS results for hnRNP isoforms.

Accession	Name	kDa	AdML-WT-PP73'UTR	AdML-WT-PP73'UTR	AdML-WT-PP73'UTR	AdML-Mut-PP73'UTR	AdML-Mut-PP7int
P38152	hnRNP G	42.3	8	11		4	9
Q00839	hnRNP U	90.6	50	57	11		
B4DLR3	hnRNP U	86.9				18	31
PO9651	hnRNP A1	38.8			7		
Q61PF2	hnRNP A1	34.2		20		10	12

Core components of the supraspliceosome were detected both in the AdML-WT and AdML-Mut assembled supraspliceosomes: The Sm proteins that are essential components of the spliceosome *in vitro* [1] and the supraspliceosome *in vivo* [12,20] were found at both supraspliceosome populations. Specific U snRNP proteins were also identified. Surprisingly, the U1 snRNP protein U1-70K, and the U2 snRNP proteins U2A' and U2B'' were not detected at the AdML-Mut assembled supraspliceosomes. The lack of detection of these proteins probably reflects lower levels of proteins in the mutant fractionation. This might be due to lower expression of AdML-Mut in the HeLa cell-lines in comparison to the AdML-WT expressing cell lines, or to lower amounts of material analyzed for the AdML-Mut assembled supraspliceosomes, or due to technical difficulties. However, the presence of these U snRNPs, as well as the tri-snRNPs were detected in both populations, as shown by the presence of other related snRNP proteins, and also by the definite Northern blot results (Figure 6) revealing the presence of the five U snRNAs, including U1 snRNA and U2 snRNA in both populations. Additional core components of the supraspliceosome found both at AdML-WT and AdML-Mut assembled supraspliceosomes is the hPRP19/CDC5L complex. The hPRP19/CDC5L complex that was previously shown to be necessary for both splicing stages [43,44,45,46] was also identified at the proteomic analysis of the general supraspliceosome population [12]. Studies *in vitro* showed that this complex joins the spliceosome together with the U4/U6.U5 tri-snRNP and remains associated with the spliceosome during both steps of splicing [38,47]. Our results for the hPRP19/CDC5L complex are consistent with the latter *in vitro* findings showing its association with the splicing complex during the splicing steps. Another group of proteins detected in both supraspliceosome populations (assembled on AdML-WT or AdML-Mut transcripts) were proteins responsible for pre-mRNA processing. These included: components of the cleavage and polyadenylation specificity factor (CPSF) that is responsible for 3' end processing [48,49,50]; editing proteins [21,51]; and the ALY/REF proteins which play a role in RNA export from the nucleus [52]. It should be pointed out that the presence of 3' end processing components [20] and the presence of ADAR (adenosine deaminases acting on RNA) enzymes [21] in supraspliceosomes was previously shown. The finding of these factors here, stresses the fact that the supraspliceosome is a machine responsible for the general RNA processing events in the cell.

The MS analyses further showed that several splicing factors and other proteins involved in RNA metabolism were associated with supraspliceosomes assembled on AdML-WT and AdML-Mut transcripts. The analyses revealed the presence of the RNA binding proteins SR and hnRNP families at both AdML-WT and AdML-Mut assembled supraspliceosomes. These proteins, which play a role in alternative and constitutive RNA splicing [52], were previously shown to be a part of the supraspliceosome [14,16,17,32]. It should be pointed out that shotgun proteomics, as was used here, is a technology that maps the composition of protein samples with an element of stochastic coverage. This is especially true for label-free quantitation, employed here. The biological replica had a wide spread, indicating additional challenges resulting from the biochemical isolation procedure. This was addressed to a level that allows discussing trends of protein composition of the WT and Mut supraspliceosomes. Regarding lack of expected factors as well as interesting hypothesis derived from candidates for specific factors one will have to wait for more in depth proteomic analyses, possibly using stable isotope labelling by amino acids in cell culture (SILAC) for better quantitation as samples are mixed early on and the biochemical isolation procedure then has no impact on the relative protein composition.

**Figure 7:**
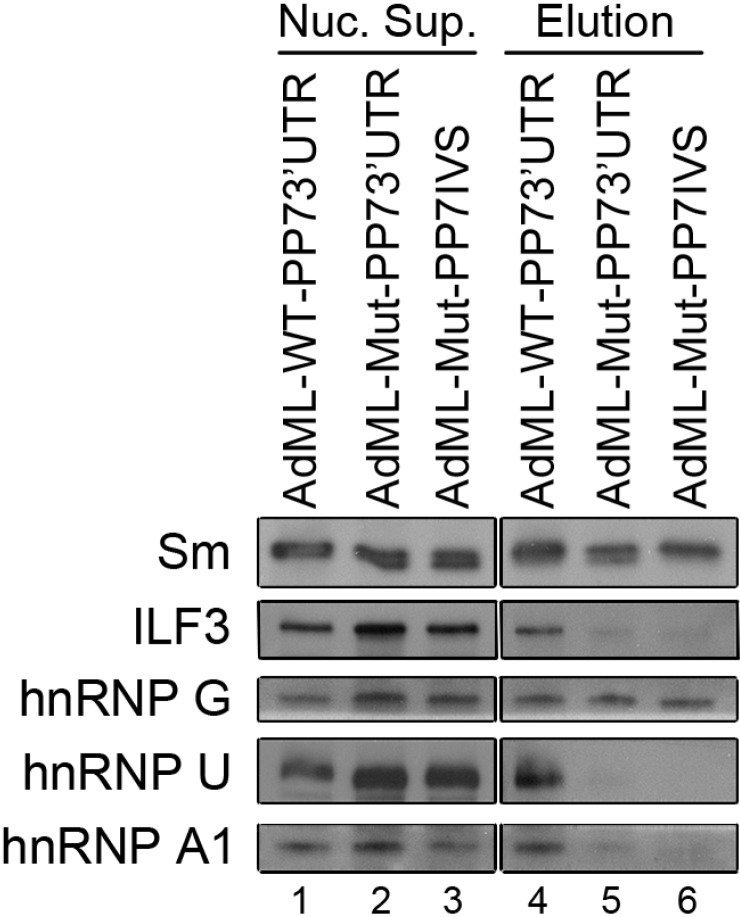
WB analyses of proteins associated with supraspliceosomes assembled on AdML-WT and AdML-Mut transcripts confirm the MS results. Nuclear supernatants were prepared from each of the HeLa cell lines, stably expressing either the AdML-WT-PP73'UTR, the AdML-Mut-PP73'UTR, or the AdML-Mut-PP7IVS transcripts, and supraspliceosomes assembled on each of the respective PP7-tagged transcripts were affinity purified, as described above. Proteins from each of the nuclear supernatants (Nuc. Sup., **left**) and from each the affinity purified supraspliceosomes (Elution, **right**) were extracted, electrophoresed on 8% polyacrylamide gel and analyzed by WB. The different antibodies used for the WB analyses are given on left.

Deeper analysis of the hnRNP proteins revealed different alternatively spliced isoforms of a number of hnRNP proteins at different stages of splicing (Table 2). While for hnRNP G, a single isoform was detected, present in both AdML-WT and AdML-Mut assembled supraspliceosomes, in the cases of hnRNP U and of hnRNP A1 proteins, two isoforms were detected. The full-length isoform of hnRNP U and of hnRNP A1 was detected only within the AdML-WT assembled supraspliceosomes, whereas the shorter isoform of hnRNP U was detected only within the AdML-Mut assembled supraspliceosomes, and the shorter isoform of hnRNP A1 was detected in both populations. Western Blot analysis of proteins associated with AdML-WT and AdML-Mut assembled supraspliceosomes (Figure 7), showed that hnRNP G is present in both complexes, similar to the Sm proteins, thus validating the MS results (Table 1). In the case of ILF3, which is involved in transcription, this 110 kDa isoform is found associated with supraspliceosomes assembled on AdML-WT transcripts and not on supraspliceosomes assembled on AdML-Mut transcripts, also in agreement with the MS results (Table 1). We also validated the MS results showing different alternatively spliced isoforms for hnRNP U and of hnRNP A1 proteins at different stages of splicing (Table 2). Thus, the full-length isoform of hnRNP U and of hnRNP A1 was detected only within the AdML-WT assembled supraspliceosomes, when analyzed by WB, using antibodies that recognize only the full-length respective protein (Figure 7).

The proteomic analyses of supraspliceosomes assembled on AdML-WT and AdML-Mut transcripts strengthen the above conclusion that the supraspliceosome is the pre-mRNA processing machine that is pre-assembled throughout all splicing stages, as the core components of the spliceosome can be seen in all these stages. The analyses also show the dynamic nature of the supraspliceosome during the splicing stages, as different hnRNP isoforms were found in supraspliceosomes from the different steps of splicing.

## 3. Experimental Section

Plasmid construction and cell culture. AdML-WT and AdML-Mut constructs in pSP72 were kindly provided by Prof. Reed R (Harvard Medical School, Boston, MA, USA). The AdML-WT and AdML-Mut fragments share identical exon 1 (70 nucleotides). While AdML-WT construct contains intron 1 (123 nucleotides) and exon 2 (51 nucleotides), the AdML-Mut construct harbors an elongated undisturbed pyrimidine tract, and a mutated 3' splice site from CAG to TGG. The second exon of AdML-Mut is essentially the same in length and sequence as in AdML-WT, except for the change in sequence of nucleotides 2–5 from TGCA to CATG. Both constructs were digested with VspI, filled in with Klenow fragment, and ligated to EcoRV digested pcDNA3 vector. The AdML-Mut construct was then further mutated (QuikChange™ site-directed mutagenesis kit; Agilent Technologies, Santa Clara, CA, USA) from CAG to CGG in order to prevent downstream exonic alternative 3' splice site using the primers 5'-TCGCGGTCTTTCCGGTACTCTTGGATCCG-3'; 5'-CGGATCCAAGAGTACCGGAAAGACCGCGA-3'.

PP7 tag was inserted to the 3'UTR of AdML-WT construct by performing Klenow reaction on the partially complementary single-stranded oligos: 5'-TTGCTAGCGCGGCCGCTAAGGAGTTTATATGGAAACCCTTAGACGTCGGCACGAGG-3'; and 5'-TTCTCGAGGGGCCCGGCACGAGTGTAGCTAAACCTCGTGCCGACGTCTAAGGG-3'. The double stranded product was then digested with ApaI and NotI, and ligated to the pcDNA-AdML-WT plasmid, digested with the same restriction enzymes, generating the pcDNA-AdML-WT-PP73'UTR plasmid, that harbors the PP7 tag at the 3'UTR of AdML.

For the insertion of the PP7 tag into the 3'UTR of AdML-Mut construct, first, annealing of the following single-stranded oligos: 5'-GGCCGCTAAGGAGTTTATATGGAAACCCTTAGGGCC-3'; and 5'-CTAAGGGTTTCCATATAAACTCCTTAGC-3' was preformed for 2 min at 98 °C, 2 additional minutes at 70 °C, and gradually cooling to 40 °C in the presence of 5 mM MgCl_2_.

Next, ligation of the resulting double stranded oligo to ApaI digested pcDNA-AdML-Mut, generated the pcDNA-pcDNA-AdML-Mut-PP73'UTR plasmid that harbors the PP7 tag at the 3'UTR of AdML. For the generation of pcDNA-AdML-Mut-PP7IVS, the pcDNA-AdML-Mut-PP73'UTR plasmid was digested with *Not*I and *Apa*I and self-ligated in order to remove the PP7 tag, followed by insertion of the double stranded oligo to the *Xho*I site of the plasmid, generating the pcDNA-AdML-Mut-PP7IVS plasmid that harbors the PP7 tag at the AdML intron. All constructs were confirmed by sequencing.

Next, five cell lines were generated, each stably expressing one of the above five constructs: (i) AdML-WT-PP73'UTR, that harbors the PP7 tag at the 3'UTR of AdML; (ii) AdML-WT, without the tag; (iii) AdML-Mut-PP73'UTR, that harbors the PP7 tag at the 3'UTR; (iv) AdML-WT-PP7IVS, that harbors the PP7 tag at the intron; and (v) AdML-Mut, without the tag. For this aim each of the above five plasmids was transfected to a HeLa cell-line using jetPEI™ transfection reagent. The cells were maintained in DMEM supplemented with 10% fetal calf serum and antibiotics for 24 h, then the DMEM was replaced to one which also contains 500 μg/mL G-418 antibiotics, allowing resistant cell colonies to appear several days afterwards. The resulting cell-lines were analyzed for the presence of the constructs by RNA extraction followed by RT-PCR and sequencing.

Protein purification. The plasmid pET28-ZZTEVPP7CP, for expressing the ZZTEVPP7CP protein was kindly provided by Prof. Collins K (University of California at Berkeley, Berkeley, CA, USA). The expression and purification of the protein was preformed as described [29].

The vector for TEV protease expression was kindly provided by Prof. David S. Waugh (National Cancer Institute at Frederick, Frederick, MD, USA) and expressed and purified at the Wolfson Centre for Applied Structural Biology, the Hebrew University of Jerusalem, Jerusalem, Israel.

Affinity purification of PP7 tagged splicing complexes. The affinity purification was performed as described [29] with some changes and without using the tobramycin step. All steps were conducted at 4 °C, with mild agitation. To 500 μL of the nuclear supernatants, originated from ~0.5–1 × 10^8^ cells, 1 μg of ZZTEVPP7CP protein was added in a solution of: 10% (*v*/*v*) glycerol, 0.1% (*v*/*v*) NP-40, 0.5 mM phenylmethylsulfonyl fluoride (PMSF), final concentration; and incubated for 90 min with rotation. Next, 250 μg of IgG agarose beads (Sigma Aldrich, Rehovot, Israel) pre-washed with binding buffer (10% (*v*/*v*) glycerol, 10 mM Tris pH 8, 2 mM MgCl_2_, 100 mM NaCl, 1 mM dl-dithiothreitol (DTT), 0.1% (*v*/*v*) NP-40, 0.5 mM PMSF and 2 mM vanadyl ribonucleoside (VR)) were added, followed by additional rotation for 90 min. Three washing steps with binding buffer and another one with TEV buffer (10% (*v*/*v*) glycerol, 10 mM Tris pH 8, 2 mM MgCl_2_, 100 mM NaCl and 1 mM DTT) were preformed for 10 min each. Elution of the bound material from the beads was performed by incubating the beads overnight with 1 μg of TEV protease in 50 μL TEV buffer. The supernatant was then transferred to a new tube. Samples were taken either for analyses or for further isolation by glycerol gradients.

Isolation of supraspliceosomes. All isolation steps were conducted at 4 °C. Nuclear supernatants enriched in supraspliceosomes were prepared as described [14,53]. Briefly, nuclear supernatants were prepared from purified cell nuclei by microsonication of the nuclei and precipitation of the chromatin in the presence of transfer RNA. The nuclear supernatant was fractionated on 10%–45% (*v*/*v*) glycerol gradients. Centrifugations were carried out at 4 °C in an SW41 rotor run at 41 krpm for 90 min (or an equivalent *w*^2^*t*). The gradients were calibrated with tobacco mosaic virus as a 200S sedimentation marker.

Protein analyses. For protein analyses, samples were acetone-precipitated, electrophoresed on SDS-polyacrylamide gels, and were either stained or blotted.

For total protein visualization by silver staining, the proteins were fixed in 40% ethanol, 10% acetic acid for 60 min, washed in double distilled water (DDW) for at least 30 min, sensitized in 0.02% Na_2_S_2_O_3_ for 1 min, and washed 3 times in DDW, 20 s each. The gel was then incubated in a solution of 0.1% AgNO_3_ and 0.074% formaldehyde for 20 min, and washed afterwards 3 times in DDW, 20 s each, and for additional 1 min. Development was performed in 3% Na_2_CO_3_, 0.02% formaldehyde, and the reaction was stopped in 5% acetic acid for 5 min.

WB analyses were performed using anti-Sm MAb Y12 [54]; anti-hnRNP U (Santa Cruz Biotechnology, Dallas, TX, USA); anti hnRNP A1 (Abcam, Tel Aviv, Israel); anti ILF3 (BD Biosciences, Franklin Lakes, NJ, USA), and visualized with horseradish peroxidase conjugated to affinity-pure Goat anti-Mouse IgG (H + L; Jackson Immunoreaserch, West Grove, PA, USA) diluted 1:3000; anti-hnRNP G kindly provided by Stefan Stamm (University of Kentucky, Lexington, KY, USA), visualized with horseradish peroxidase conjugated to affinity-pure Goat anti-Rabbit IgG (H + L; Jackson Immunoreaserch) diluted 1:10,000.

For mass spectrometry analyses, the proteins were acetone-precipitated, electrophoresed 1 mm into NuPAGE 4%–12% Bis-Tris gel (Life Technologies, Modiin, Israel), and stained using the colloidal blue staining kit (Life Technologies), according to the manufacturer’s instructions. Each sample corresponded to a single band of coomassie-stained gel and was excised. The proteins where digested using Trypsin as described elsewhere [55]. In brief, proteins were reduced in 10 mM DTT for 30 min at 37 °C, alkylated in 55 mM iodoacetamide for 20 min at room temperature in the dark, and digested overnight at 37 °C with 12.5 ng/μL Trypsin (Proteomics Grade, Sigma, St. Louis, MO, USA). The digestion solution was then acidified to 0.1% of TFA and spun onto StageTips as described in the literature [56]. Peptides were eluted in 20 μL of 80% acetonitrile in 0.1% TFA and were concentrated to 4 μL (Concentrator 5301, Eppendorf AG, Hamburg, Germany). The peptides sample was then diluted to 5 μL by 0.1% TFA for LC-MS/MS analysis.

Analyses were performed using a LTQ-Orbitrap mass spectrometer (Thermofisher Scientific, Waltham, MA, USA) coupled on-line to an Agilent 1200 binary nano pump (Palo Alto, CA, USA) and an HTC PAL autosampler (CTC, Zwingen, Switzerland). The analytical column with a self-assembled particle frit [57] and C18 material (ReproSil-Pur C18-AQ 3 μm; Dr. Maisch, GmbH, Ammerbuch, Germany) was packed into a spray emitter (75-μm ID, 8-μm opening, 300-mm length; New Objective, Woburn, MA, USA) using an air-pressure pump (Proxeon Biosystems, Vienna, Austria). Mobile phase A consisted of 5% acetonitrile and 0.5% acetic acid in water; mobile phase B, consisted of 0.5% acetic acid in acetonitrile. The peptides were loaded onto the column at a flow rate of 0.7 μL/min and eluted at a flow rate of 0.3 μL/min using a three-step linear gradient raising to 5% buffer B in 1 min, followed by increase to 23% buffer B in 135 min and then to 80% buffer B in 10 min.

FTMS spectra were recorded at 60,000 resolution and the six most intense peaks of the MS scan were selected in the ion trap for MS2, (normal scan, wideband activation, filling 5.0 × 10^5^ ions for MS scan, 1.0 × 10^4^ ions for MS2, maximum fill time 100 ms, dynamic exclusion for 180 s). Searches were conducted using Mascot against a database containing human sequences [58]. The search parameters were: MS accuracy, 6 ppm; MS/MS accuracy, 0.6 Da; enzyme, trypsin; allowed number of missed cleavages, 2; fixed modification, carbamidometylation on Cysteine; variable modification, oxidation on Methionine.

RNA isolation and RT-PCR analyses. For RNA extraction, samples (up to 250 μL) in 10% (*v*/*v*) glycerol, 10 mM Tris pH 8, 100 mM NaCl, 2 mM MgCl_2_ and 2 mM VR were mixed with 75 μL of extraction buffer (50 mM Tris pH 7.5, 150 mM NaCl) and 25 μL of 10% (*w*/*v*) SDS. The RNA was recovered by extraction with phenol and precipitation in ethanol. The RNA was treated with DNase I (50 U/mL; Promega, Madison, WI, USA). cDNA was synthesized from up to 3 µg of RNA, using dT15 primer and MMLV reverse transcriptase (Promega). PCRs (20 µL) containing 2.5 µL of the cDNA products, 10 pmol of the relevant primers and 1 U of Taq DNA polymerase (Promega) were preformed. The following primer pairs were used: Actin: 5'-CAAGGCCAACCGCGAGAAGATGAC-3' and 5'-AGGAAGGAAGGCTGGAAGAGTGC-3'; AdML: 5'-AATTCGAGCTCGGTACCCC-3' or 5'-GTTCGTCCTCACTCTCTT-3' and 5'-GAGGGGCAAACAACAGATGG-3'.

Electron microscopy. 10 μL aliquots of the samples were absorbed on glow-discharged carbon-coated grids and negatively stained with 1% uranyl acetate. A Tecnai 12 TEM (FEI, Eindhoven, The Netherlands), operating at acceleration voltage of 100 kV, equipped with a 1 K × 1 K TEMcam CCD camera was used (TVIPS, Gauting, Germany).

U snRNA analysis. For Northern blot analysis, RNA was extracted from the nuclear supernatants or from the affinity purified splicing complexes prepared from the stable cell lines described above, and electrophoresed on a 7 M urea/10% polyacrylamide gel. The RNA was then transferred to a Hybond-XL membrane (GE healthcare, Pittsburgh, PA, USA) and hybridized with [α-^32^P] UTP-labeled RNA transcripts, each complementary to either U1, U2, U4, U5, and U6 snRNAs as described [15,16], with minor changes. Briefly, the U snRNA plasmids were linearized, extracted with phenol and precipitated in ethanol. 1 μg from each plasmid was used as template for *in vitro* splicing reaction (20 μL), which also contained 0.5 mM ATP, 0.5 mM CTP, 5 mM GTP, 0.25 mM UTP, 40 mM Tris pH 7.5, 6 mM MgCl_2_, 10 mM DTT, 10 mM NaCl, 2 mM spermidine, 50 U RNasin (EURX, Gdansk, Poland), 20 μCi of [α-^32^P] UTP (PerkinElmer, 3000 Ci/nmol) and 20 U of RNA polymerase (Promega). The reaction was preformed for 90 min at 37 °C, and cleaned with Sephadex G-25 beads (Sigma Aldrich, St. Louis, MO, USA). Hybridization was performed by overnight incubation of the membrane, at 65 °C, with 1.5 × 10^7^ cpm mix of the five RNA probes in 10 mL hybridization buffer (340 mM Na_2_HPO_4_, 160 mM NaH_2_PO_4_, 1% BSA, 7% SDS, 1 mM EDTA). The membrane was then washed with 3 consecutive washing buffers [washing buffer #1 (2× SSC, 0.1% SDS); washing buffer #2 (1× SSC, 0.1% SDS); washing buffer #3 (0.1× SSC, 0.2% SDS)], for 15 min each. Washing with washing buffer 1 was performed at room temperature. Washing with buffers 2 and 3 were performed at 65 °C. The membrane was exposed to a phosphorimager. Bands were quantified with Image Gauge version 3.46 software (FUJIFILM, Benei Brak, Israel).

## 4. Conclusions

Isolation and characterization of splicing complexes assembled *in vivo* on specific transcripts, at defined functional states, revealed that they are assembled in supraspliceosomes at all splicing stages. This study also demonstrated that in cell nuclei, transcripts having one intron are also assembled and processed in supraspliceosomes. This is in accordance with previous experiments with reconstituted supraspliceosomes, which showed that pre-mRNAs with less than four introns are also assembled in supraspliceosomes [15]. Likely, the interactions of the RNA with the substructures of the supraspliceosome are sufficient to hold the structure together [15]. Analysis of the U snRNA composition of these supraspliceosomes revealed that they are assembled with all five U snRNAs at all splicing stages. We can, thus, conclude that dynamic changes in base-pairing interactions of U snRNA:U snRNA and U snRNA:pre-mRNA that occur *in vivo* during the splicing reaction do not require changes in U snRNP composition of the supraspliceosome. We can further conclude that supraspliceosomes isolated from mammalian cell nuclei are pre-assembled splicing complexes at all splicing stages. Furthermore, there is no need to reassemble a native spliceosome for the splicing of each intron, and rearrangements of the interactions will suffice. Protein composition analyses of specific supraspliceosomes assembled on mature and precursor molecules show that the core splicing components are present at all splicing stages, supporting the pre-assembled nature of the supraspliceosome. The dynamic nature of the splicing machine was also shown, as some differences in protein composition were observed in supraspliceosomes at the different splicing stages. The finding of RNA processing components, such as 3' end processing and RNA editing, as integral components of supraspliceosomes further support the notion that the supraspliceosome is the nuclear pre-mRNA processing machine.

## Supplementary Files

Supplementary File 1
