# Supplementary material for: Supraspliceosomes at Defined Functional States Portray the Pre-Assembled Nature of the Pre-mRNA Processing Machine in the Cell Nucleus
Source: Int J Mol Sci. 2014 Jun 30;15(7):11637–64. doi: 10.3390/ijms150711637 (PMC4139805; doi:10.3390/ijms150711637)
Supplement: Supplementary File 1 [file ijms-15-11637-s001.pdf]

Supplementary Information

Table S1. MS analyses.

Accession	Symbol	Name	Mass (Da)	AdML-WT-PP73'UTR	AdML-WT-PP73'UTR	AdML-WT-PP73'UTR	AdML-Mut-PP73'UTR	AdML-Mut-PP7IVS
			Sm Proteins					
A8MWD9	–	Small nuclear ribonucleoprotein G-like protein	8544	2	3	–	2	–
B4DVS0	SNRPB	Small nuclear ribonucleoprotein-associated protein	24,088	–	–	–	5	–
P14678	SNRPB	Small nuclear ribonucleoprotein-associated proteins B and B'	24,610	–	–	2	–	–
P62314	SNRPD1	Small nuclear ribonucleoprotein Sm D1	13,282	3	5	1	3	–
J3QLI9	SNRPD1	Small nuclear ribonucleoprotein Sm D1	8393	–	–	–	–	2
P62316	SNRPD2	Small nuclear ribonucleoprotein Sm D2	13,527	2	3	1	–	–
P62318	SNRPD3	Small nuclear ribonucleoprotein Sm D3	13,916	–	–	2	–	–
B4DJP7	SNRPD3	Small nuclear ribonucleoprotein Sm D3	13,292	4	4	–	3	3
P62304	SNRPE	Small nuclear ribonucleoprotein E	10,804	2	3	2	4	1
B3KVR1	SNRPN	Small nuclear ribonucleoprotein-associated protein	25,076	5	7	–	–	4
			U1 snRNP					
A8KAQ5	–	cDNA FLJ77404, highly similar to Homo sapiens small nuclear ribonucleoprotein 70 kDa polypeptide (RNP antigen) (SNRP70), transcript variant 1, mRNA	51,496	–	5	–	–	–
			U2 snRNP					
B3KY12	–	cDNA FLJ46581 fis, clone THYMU3043200, highly similar to Splicing factor 3A subunit 3	58,777	12	10	–	1	2
A8K6V3	–	cDNA FLJ78677, highly similar to Homo sapiens splicing factor 3b, subunit 3, 130 kDa (SF3B3), mRNA	135,578	–	–	–	8	–
Q53G21	–	Small nuclear ribonucleoprotein polypeptide A' variant	28,474	9	7	–	–	–
Q7L014	DDX46	Probable ATP-dependent RNA helicase DDX46	117,362	1	4	–	–	1
Q7RTV0	PHF5A	PHD finger-like domain-containing protein 5A	12,405	2	1	1	1	–
Q15459	SF3A1	Splicing factor 3A subunit 1	88,886	22	17	7	–	–
Q15428	SF3A2	Splicing factor 3A subunit 2	49,256	–	–	2	–	–

Table S1. *Cont.*

Accession	Symbol	Name	Mass (Da)	AdML-WT-PP73'UTR	AdML-WT-PP73'UTR	AdML-WT-PP73'UTR	AdML-Mut-PP73'UTR	AdML-Mut-PP7IVS
		U2 snRNP						
Q05DF2	SF3A2	SF3A2 protein	51,476	10	7	–	–	–
Q12874	SF3A3	Splicing factor 3A subunit 3	58,849	–	–	8	–	–
O75533	SF3B1	Splicing factor 3B subunit 1	145,830	26	5	–	5	–
H0YCG1	SF3B2	Splicing factor 3B subunit 2	42,457	–	–	–	–	2
Q13435	SF3B2	Splicing factor 3B subunit 2	100,228	20	12	3	3	1
Q15393	SF3B3	Splicing factor 3B subunit 3	135,577	30	23	8	–	–
P09661	SNRPA1	U2 small nuclear ribonucleoprotein A'	28,416	–	–	2	–	–
P08579	SNRPB2	U2 small nuclear ribonucleoprotein B''	25,486	3	4	1	–	–
		U2 snRNP associated						
B5BU25	U2AF2	U2 small nuclear RNA auxiliary factor 2 isoform b	53,137	3	–	–	2	–
K7ENG2	U2AF2	Splicing factor U2AF 65 kDa subunit	33,902	–	–	–	–	4
P26368	U2AF2	Splicing factor U2AF 65 kDa subunit	53,501	–	5	–	–	–
		U4/U6.U5 snRNP						
B2R791	–	cDNA, FLJ93335, highly similar to Homo sapiens PRP3 pre-mRNA processing factor 3 homolog (yeast) (PRPF3), mRNA	77,543	1	3	–	–	–
B2R7V4	–	cDNA, FLJ93619, highly similar to Homo sapiens PRP4 pre-mRNA processing factor 4 homolog (yeast) (PRPF4), mRNA	58,449	6	4	–	1	2
B3KX19	–	cDNA FLJ44500 fis, clone UTERU3000828, highly similar to 116 kDa U5 small nuclear ribonucleoprotein component	108,210	6	–	–	6	–
O94906	PRPF6	Pre-mRNA-processing factor 6	106,925	–	–	–	3	–
Q6P2Q9	PRPF8	Pre-mRNA-processing-splicing factor 8	273,600	9	19	–	10	–
F1T0A5	PRPF31	PRP31 pre-mRNA processing factor 31 homolog (Yeast), isoform CRA a	55,456	–	2	–	–	–
O75643	SNRNP200	U5 small nuclear ribonucleoprotein 200 kDa helicase	244,508	10	–	–	–	–

Table S1. *Cont.*

Accession	Symbol	Name	Mass (Da)	AdML-WT-PP73'UTR	AdML-WT-PP73'UTR	AdML-WT-PP73'UTR	AdML-Mut-PP73'UTR	AdML-Mut-PP7IVS
A4FU77	SNRNP200	SNRNP200 protein	216,183	–	14	–	5	5
Q6IBM8	U5-116KD	U5-116KD protein	109,460	–	11	–	–	–
B3KY11	–	cDNA FLJ46571 fis, clone THYMU3041428, highly similar to Probable ATP-dependent RNA helicase DDX23 (EC 3.6.1.-)	93,234	–	2	–	2	2
hPRP19/CDC5L complex								
B3KY60	–	cDNA FLJ16777 fis, clone BRHIP2029567, highly similar to Cell division cycle 5-like protein	92,277	5	6	–	–	–
P11142	HSPA8	Heat shock cognate 71 kDa protein	70,898	22	20	9	15	18
A8MW61	PLRG1	Pleiotropic regulator 1	57,182	–	2	–	–	–
Q9UMS4	PRPF19	Pre-mRNA-processing factor 19	55,181	8	9	2	4	2
F5H315	XAB2	Pre-mRNA-splicing factor SYF1	99,680	–	2	–	–	–
hnRNPs								
B2R5W2	–	cDNA, FLJ92657, highly similar to Homo sapiens heterogeneous nuclear ribonucleoprotein C (C1/C2) (HNRPC), transcript variant 2, mRNA	31,948	20	24	–	12	9
B2R7W4	–	cDNA, FLJ93632, highly similar to Homo sapiens heterogeneous nuclear ribonucleoprotein R (HNRPR), mRNA	70,902	–	–	–	–	6
B4DMY3	–	cDNA FLJ60713, highly similar to Homo sapiens heterogeneous nuclear ribonucleoprotein A/B (HNRPAB), transcript variant 1, mRNA	35,009	17	12	–	6	1
B4DTA2	–	cDNA FLJ60148, highly similar to Homo sapiens heterogeneous nuclear ribonucleoprotein D-like (HNRPDL), transcript variant 2, mRNA	30,214	–	–	–	–	2
Q53F48	–	Heterogeneous nuclear ribonucleoprotein H3 isoform a variant	36,926	1	–	–	4	5

Table S1. *Cont.*

Accession	Symbol	Name	Mass (Da)	AdML-WT-PP73'UTR	AdML-WT-PP73'UTR	AdML-WT-PP73'UTR	AdML-Mut-PP73'UTR	AdML-Mut-PP7IVS
A8K3W4	–	cDNA FLJ75163, highly similar to Homo sapiens heterogeneous nuclear ribonucleoprotein U-like 1 (HNRPUL1), transcript variant 4, mRNA	84,822	–	4	–	–	–
B2R8Z8	–	cDNA, FLJ94136, highly similar to Homo sapiens synaptotagmin binding, cytoplasmic RNA interacting protein (SYNCRIP), mRNA	69,602	–	–	–	3	–
B4DKS8	–	cDNA FLJ57121, highly similar to Heterogeneous nuclear ribonucleoprotein F	37,255	6	–	–	3	1
B4DLR3	–	cDNA FLJ54020, highly similar to Heterogeneous nuclear ribonucleoprotein U	86,861	–	–	–	31	18
B4DUQ1	–	cDNA FLJ54552, highly similar to Heterogeneous nuclear ribonucleoprotein K	48,510	31	–	–	–	11
Q13151	HNRNPA0	Heterogeneous nuclear ribonucleoprotein A0	30,841	4	5	2	4	5
F8VRQ1	HNRNPA1	Heterogeneous nuclear ribonucleoprotein A1	33,155	19	–	–	–	–
P09651	HNRNPA1	Heterogeneous nuclear ribonucleoprotein A1	38,747	–	–	7	–	–
P22626	HNRNPA2B1	Heterogeneous nuclear ribonucleoproteins A2/B1	37,430	15	20	10	18	16
P51991	HNRNPA3	Heterogeneous nuclear ribonucleoprotein A3	39,595	–	14	3	–	–
P07910	HNRNPC	Heterogeneous nuclear ribonucleoproteins C1/C2	33,670	–	–	6	–	–
H0Y8G5	HNRNPD	Heterogeneous nuclear ribonucleoprotein D0	29,724	–	15	–	–	–
Q14103	HNRNPD	Heterogeneous nuclear ribonucleoprotein D0	38,434	12	–	4	–	–
P52597	HNRNPF	Heterogeneous nuclear ribonucleoprotein F	45,672	–	5	5	–	–
P31943	HNRNPH1	Heterogeneous nuclear ribonucleoprotein H	49,229	–	–	8	–	–
G8JLB6	HNRNPH1	Heterogeneous nuclear ribonucleoprotein H	51,230	13	13	–	9	11
P55795	HNRNPH2	Heterogeneous nuclear ribonucleoprotein H2	49,264	3	2	–	3	–
P31942	HNRNPH3	Heterogeneous nuclear ribonucleoprotein H3	36,926	–	6	–	–	–
P61978	HNRNPK	Heterogeneous nuclear ribonucleoprotein K	50,976	–	29	14	23	–
P14866	HNRNPL	Heterogeneous nuclear ribonucleoprotein L	64,133	–	–	3	–	–

Table S1. *Cont.*

Accession	Symbol	Name	Mass (Da)	AdML-WT-PP73'UTR	AdML-WT-PP73'UTR	AdML-WT-PP73'UTR	AdML-Mut-PP73'UTR	AdML-Mut-PP7IVS
Q6NTA2	HNRNPL	HNRNPL protein	61,927	28	23	–	17	5
P52272	HNRNPM	Heterogeneous nuclear ribonucleoprotein M	77,516	28	35	2	14	12
O43390	HNRNPR	Heterogeneous nuclear ribonucleoprotein R	70,943	27	32	3	19	–
Q00839	HNRNPU	Heterogeneous nuclear ribonucleoprotein U	90,584	50	57	11	–	–
B7Z4B8	HNRNPUL1	Heterogeneous nuclear ribonucleoprotein U-like protein 1	86,122	–	–	–	8	4
Q9BUJ2	HNRNPUL1	Heterogeneous nuclear ribonucleoprotein U-like protein 1	95,739	–	–	4	–	–
Q1KMD3	HNRNPUL2	Heterogeneous nuclear ribonucleoprotein U-like protein 2	85,105	21	19	1	7	1
Q6IPF2	HNRPA1	Heterogeneous nuclear ribonucleoprotein A1	34,180	–	20	–	12	10
B4DDB6	HNRPA3	Heterogeneous nuclear ribonucleoprotein A3, isoform CRA a	37,029	13	–	–	5	4
O14979	HNRPDL	Heterogeneous nuclear ribonucleoprotein D-like	46,438	16	9	6	9	–
O60506	SYNCRIP	Heterogeneous nuclear ribonucleoprotein Q	69,603	17	8	4	–	–
Q53SS8	PCBP1	Poly(RC) binding protein 1	37,498	5	3	–	1	–
B4DXP5	PCBP2	Poly(rC)-binding protein 2	33,926	2	–	–	–	–
F8VXH9	PCBP2	Poly(rC)-binding protein 2	16,997	–	2	–	–	–
P26599	PTBP1	Polypyrimidine tract-binding protein 1	57,221	15	11	4	4	–
Q5QPL9	RALY	RNA binding protein, autoantigenic (HnRNP-associated with lethal yellow homolog (Mouse)	24,665	6	11	–	–	1
P38159	RBMX	RNA-binding motif protein, X chromosome	42,332	8	11	–	9	4

Table S1. *Cont.*

Accession	Symbol	Name	Mass (Da)	AdML-WT-PP73'UTR	AdML-WT-PP73'UTR	AdML-WT-PP73'UTR	AdML-Mut-PP73'UTR	AdML-Mut-PP7IVS
SR proteins								
A8K588	–	cDNA FLJ76823, highly similar to Homo sapiens splicing factor, arginine/serine-rich 6 (SFRS6), mRNA	39,488	–	3	–	–	3
B2RDQ3	–	cDNA, FLJ96718, highly similar to Homo sapiens splicing factor, arginine/serine-rich 10 (transformer 2 homolog, Drosophila) (SFRS10), mRNA	33,682	2	2	–	1	1
B3KUF7	–	cDNA FLJ39750 fis, clone SMINT2017736, moderately similar to SPLICING FACTOR, ARGININE/SERINE-RICH 2	21,352	–	–	–	–	2
B4DEK2	–	cDNA FLJ59182, highly similar to Splicing factor, arginine/serine-rich 7	18,815	3	3	–	2	3
B2R6F3	SFRS3	Splicing factor arginine/serine-rich 3	19,330	3	–	–	2	3
Q05BU6	SFRS11	SFRS11 protein	24,847	–	2	–	–	–
Q9UQ35	SRRM2	Serine/arginine repetitive matrix protein 2	299,615	2	–	–	–	–
J3KTL2	SRSF1	Serine/arginine-rich-splicing factor 1	28,329	6	6	–	2	3
B4E241	SRSF3	Serine/arginine-rich-splicing factor 3	14,203	–	4	–	–	–
Splicing factors								
B4E0S6	–	cDNA FLJ55635, highly similar to pre-mRNA-splicing factorATP-dependent RNA helicase DHX15 (EC 3.6.1.-)	89,547	6	3	–	8	2
J3KTA4	DDX5	Probable ATP-dependent RNA helicase DDX5	69,087	13	15	–	15	11
H3BPE7	FUS	RNA-binding protein FUS	53,497	–	–	–	5	5
P35637	FUS	RNA-binding protein FUS	53,426	–	–	3	–	–
Q13344	–	Fus-like protein	53,377	2	–	–	–	–
O00148	DDX39A	ATP-dependent RNA helicase DDX39A	49,130	13	5	–	–	–
F8VQ10	DDX39B	Spliceosome RNA helicase DDX39B	50,745	4	13	–	–	10

Table S1. *Cont.*

Accession	Symbol	Name	Mass (Da)	AdML-WT-PP73'UTR	AdML-WT-PP73'UTR	AdML-WT-PP73'UTR	AdML-Mut-PP73'UTR	AdML-Mut-PP7IVS
B4DX78	–	cDNA FLJ55484, highly similar to ATP-dependent RNA helicase DDX39 (EC 3.6.1.-)	53,697	–	–	5	5	1
Q92945	KHSRP	Far upstream element-binding protein 2	73,115	4	1	–	–	–
P11940	PABPC1	Polyadenylate-binding protein 1	70,671	19	19	8	18	–
Q9BSV4	SFPQ	SFPQ protein	68,631	–	3	–	6	5
P67809	YBX1	Nuclease-sensitive element-binding protein 1	35,924	–	8	3	–	–
Q05D43	YBX1	YBX1 protein	29,505	5	–	–	4	–
Q9UHX1	PUF60	Poly(U)-binding-splicing factor PUF60	59,875	1	3	–	–	–
B4DJ45	TARDBP	TAR DNA-binding protein 43	31,808	–	2	–	–	–
Q5BKZ1	ZNF326	DBIRD complex subunit ZNF326	65,654	2	3	–	1	–
A0MNN4	SMU1	CDW3/SMU1	57,544	–	3	–	–	–
Q8N163	KIAA1967	DBIRD complex subunit KIAA1967	102,902	–	–	4	–	–
A8K525	–	cDNA FLJ76817, highly similar to Homo sapiens non-POU domain containing, octamer-binding (NONO), mRNA	54,288	–	3	–	5	9
A8K9U0	–	cDNA FLJ78260, highly similar to Homo sapiens RNA binding motif protein 4, mRNA	40,284	–	3	–	–	–
C9JTN7	TIA1	Nucleolysin TIA-1 isoform p40	31,625	5	–	–	–	–
3' polyA/processing factors								
B2R6U8	–	cDNA, FLJ93125, highly similar to Homo sapiens cleavage and polyadenylation specific factor 5, 25 kDa(CPSF5), mRNA	26,215	3	3	–	2	5
B3KMI0	–	cDNA FLJ11050 fis, clone PLACE1004564, highly similar to Cleavage and polyadenylation specificity factor 100 kDa subunit	73,014	–	3	–	–	–
B4DGF8	–	cDNA FLJ57877, highly similar to Cleavage and polyadenylation specificity factor 7	51,106	2	2	–	1	–

Table S1. *Cont.*

Accession	Symbol	Name	Mass (Da)	AdML-WT-PP73'UTR	AdML-WT-PP73'UTR	AdML-WT-PP73'UTR	AdML-Mut-PP73'UTR	AdML-Mut-PP7IVS
D3DWL9	CPSF1	Cleavage and polyadenylation specific factor 1, 160kDa, isoform CRA a	151,986	8	2	–	–	–
G5E9W3	CPSF3	Cleavage and polyadenylation specific factor 3, 73kDa, isoform CRA b	73,477	5	2	–	–	–
F8WJN3	CPSF6	Cleavage and polyadenylation-specificity factor subunit 6	52,270	5	6	–	2	1
E9PID8	CSTF2	Cleavage stimulation factor subunit 2	46,666	2	3	–	–	–
Q9P2I0	CPSF2	Cleavage and polyadenylation specificity factor subunit 2	88,487	6	–	–	–	–
O43809	NUDT21	Cleavage and polyadenylation specificity factor subunit 5	26,227	–	–	2	–	–
H0YJH9	PABPN1	Polyadenylate-binding protein 2	11,083	2	3	–	1	–
B7Z6F7	–	cDNA FLJ61705, highly similar to Symplekin	141,132	6	–	–	–	–
		Cap binding						
Q09161	NCBP1	Nuclear cap-binding protein subunit 1	91,839	3	1	1	–	–
		Editing						
E7ENU4	ADAR	Double-stranded RNA-specific adenosine deaminase	140,828	10	–	–	3	–
H0YCK3	ADAR	Double-stranded RNA-specific adenosine deaminase	132,647	–	6	–	–	–
		mRNA export and surveillance						
E9PB61	ALYREF	THO complex subunit 4	27,558	–	4	–	–	–
Q92900	UPF1	Regulator of nonsense transcripts 1	124,345	6	6	–	2	–
Q96A72	MAGOHB	Protein mago nashi homolog 2	17,276	–	5	–	–	–
Q9NZI8	IGF2BP1	Insulin-like growth factor 2 mRNA-binding protein 1	63,481	2	2	–	–	–
H0YFC6	RAN	GTP-binding nuclear protein Ran	11,680	–	–	–	–	2
J3KQE5	RAN	GTP-binding nuclear protein Ran	26,816	8	–	–	1	–
P62826	RAN	GTP-binding nuclear protein Ran	24,423	–	–	2	–	–
O14980	XPO1	Exportin-1	123,386	5	–	–	–	–

Table S1. *Cont.*

Accession	Symbol	Name	Mass (Da)	AdML-WT-PP73'UTR	AdML-WT-PP73'UTR	AdML-WT-PP73'UTR	AdML-Mut-PP73'UTR	AdML-Mut-PP7IVS
P49792	RANBP2	E3 SUMO-protein ligase RanBP2	358,199	26	–	1	5	3
P46060	RANGAP1	Ran GTPase-activating protein 1	63,542	11	4	3	–	–
P38919	EIF4A3	Eukaryotic initiation factor 4A-III	46,871	2	6	–	2	–
B5BTY4	DDX3X	ATP-dependent RNA helicase DDX3X	73,171	3	5	–	8	8
RNA metabolism								
B1AMU3	RP11-452K12.9	Exosomal core protein CSL4	18,817	2	–	–	–	–
Q13868	EXOSC2	Exosome complex component RRP4	32,789	–	–	2	–	–
A8QI98	–	DIS3	109,058	4	1	–	–	–
P42285	SKIV2L2	Superkiller viralicidic activity 2-like 2	117,805	11	–	–	–	–
B4E3M6	–	cDNA FLJ55446, highly similar to Superkiller viralicidic activity 2-like 2 (EC 3.6.1.-)	106,757	–	3	–	2	–
Q8NC51	SERBP1	Plasminogen activator inhibitor 1 RNA-binding protein	44,965	–	9	–	–	–
Q96SI9	STRBP	Spermatid perinuclear RNA-binding protein	73,653	7	4	–	–	–
A3RJH1	DDX1	ATP-dependent RNA helicase DDX1	82,432	8	8	–	–	–
F1T0B3	DDX1	ATP-dependent RNA helicase DDX1	73,915	–	–	–	5	1
Q9NR30	DDX21	Nucleolar RNA helicase 2	87,344	3	4	–	8	2
Q01085	TIAL1	Nucleolysin TIAR	41,591	–	–	4	–	–
B3KM80	NCL	Nucleolin, isoform CRA c	58,554	–	–	–	–	2
P19338	NCL	Nucleolin	76,614	33	–	–	–	–
Q6ZS99	–	cDNA FLJ45706 fis, clone FEBRA2028457, highly similar to Nucleolin	65,962	–	14	3	3	–
P06748	NPM1	Nucleophosmin	32,575	14	20	12	–	7
Q9BTI9	NPM1	NPM1 protein	25,049	–	–	–	2	–
B3KWX7	–	cDNA FLJ44170 fis, clone THYMU2035319, highly similar to RNA-binding region-containing protein 2	56,744	4	–	–	–	–
B1ANR0	PABPC4	Poly(A) binding protein, cytoplasmic 4 (Inducible form)	67,971	6	8	–	1	3

Table S1. *Cont.*

Accession	Symbol	Name	Mass (Da)	AdML-WT-PP73'UTR	AdML-WT-PP73'UTR	AdML-WT-PP73'UTR	AdML-Mut-PP73'UTR	AdML-Mut-PP7IVS
Q13310	PABPC4	Polyadenylate-binding protein 4	70,783	–	–	2	–	–
Q12905	ILF2	Interleukin enhancer-binding factor 2	43,062	–	–	9	–	–
B4DY09	–	cDNA FLJ51660, highly similar to Interleukin enhancer-binding factor 2	38,910	19	18	–	6	2
G5E9M5	ILF3	Interleukin enhancer binding factor 3, 90 kDa, isoform CRA b	95,808	1	34	–	–	–
Q12906	ILF3	Interleukin enhancer-binding factor 3	95,338	–	–	13	–	–
A8K590	–	cDNA FLJ77456, highly similar to Homo sapiens interleukin enhancer binding factor 3, 90 kDa (ILF3), transcript variant 2, mRNA	76,051	44	–	–	–	–
F4ZW64	–	NF90a	75,961	–	–	–	10	–
F4ZW65	–	NF90b	76,472	–	–	–	–	5
B4DVB8	ELAVL1	ELAV-like protein 1	38,996	21	19	7	4	4
A8MXP9	MATR3	Matrin-3	99,967	20	24	–	15	11
P43243	MATR3	Matrin-3	94,623	–	–	2	–	–
E5KNY5	LRPPRC	Leucine-rich PPR-motif containing	157,905	24	5	–	–	–
B7ZKM0	SART3	SART3 protein	105,584	7	–	–	–	–
H0Y2W2	ATAD3A	ATPase family AAA domain-containing protein 3A	64,356	–	–	–	–	3
Q9NVI7	ATAD3A	ATPase family AAA domain-containing protein 3A	71,369	–	–	–	8	–
